# Numerical Study of Bubble Breakup in Fractal Tree-Shaped Microchannels

**DOI:** 10.3390/ijms20215516

**Published:** 2019-11-05

**Authors:** Chengbin Zhang, Xuan Zhang, Qianwen Li, Liangyu Wu

**Affiliations:** 1 Key Laboratory of Energy Thermal Conversion and Control of Ministry of Education, School of Energy and Environment, Southeast University, Nanjing, Jiangsu 210096, China; cbzhang@seu.edu.cn (C.Z.); xzhang@microflows.net (X.Z.); qwli@microflows.net (Q.L.); 2 College of Electrical, Energy and Power Engineering, Yangzhou University, Yangzhou 225127, China

**Keywords:** bubble, break up, fractal, tree, microchannel

## Abstract

Hydrodynamic behaviors of bubble stream flow in fractal tree-shaped microchannels is investigated numerically based on a two-dimensional volume of fluid (VOF) method. Bubble breakup is examined in each level of bifurcation and the transition of breakup regimes is discussed in particular. The pressure variations at the center of different levels of bifurcations are analyzed in an effort to gain further insight into the underlying mechanism of bubble breakup affected by multi-levels of bifurcations in tree-shaped microchannel. The results indicate that due to the structure of the fractal tree-shaped microchannel, both lengths of bubbles and local capillary numbers decrease along the microchannel under a constant inlet capillary number. Hence the transition from the obstructed breakup and obstructed-tunnel combined breakup to coalescence breakup is observed when the bubbles are flowing into a higher level of bifurcations. Compared with the breakup of the bubbles in the higher level of bifurcations, the behaviors of bubbles show stronger periodicity in the lower level of bifurcations. Perturbations grow and magnify along the flow direction and the flow field becomes more chaotic at higher level of bifurcations. Besides, the feedback from the unequal downstream pressure to the upstream lower level of bifurcations affects the bubble breakup and enhances the upstream asymmetrical behaviors.

## 1. Introduction

Segmented bubble in gas–liquid multiphase flow is ubiquitous in a variety of applications such as sample analysis [1,2,3], sorting [4], mixing [5] and heat transfer [6,7] in microfluidics. In particular, the active ingredients can be encapsulated inside a bubble and released to the target precisely, which makes the bubbles highly desirable in drug delivery systems [8,9,10,11]. Since bubbles are usually immiscible with the carrying of continuous fluid, complex interfacial phenomena are encountered during the flow of bubbles [12]. For example, breakup and splitting of bubbles are observed in T-junctions [13], while deformation is observed when the bubble is subjected to Couette flow [14]. The dynamic behaviors of bubbles are strongly affected by the local geometry [15], which is significant to the efficiency of treatment, especially when bubbles are acting as drug delivery vessels. It is worth noting that bifurcations are the most common structures in human transportation systems, which is essential to understanding the dynamics of bubbles flowing in bifurcations for the optimal design of delivering systems.

During the past decades, bubble flow in bifurcation has been widely investigated through both experimental and numerical approaches [16,17,18], among which the breakup of a single bubble or bubble streams in a single T-junction are mostly studied. Combined with a high-speed camera and a micro-particle image velocimetry (micro-PIV) system, the breakup of bubbles is examined visually by Fu et al. [13] in a PMMA (polymethyl methacrylate) square channel with the capillary number ranging between 0.001 and 0.1. Three symmetrical breakup regimes are distinguished and the transition between breakup and non-break is examined. In particular, a scaling law is found to predict the variation of the minimum bubble neck during the extension of bubbles inside the branches. The results also suggest that the behaviors of bubble and droplet in microfluidic devices are similar. The critical length of bubble breaking in an asymmetric T-junction is examined by Wang et al. [19] experimentally in an effort to examine the dominate dimensionless functions. The transition between the permanent obstructed breakup and partly permanent obstructed breakup is determined by two different dimensionless bubble lengths. While the transition between the partly obstructed breakup and tunnel break is found to be only dependent on one dimensionless bubble length. Numerical efforts have also been conducted to provide further insight into the transit features of bubble breakup using a volume of fluid (VOF) method by He et al. [20]. Coalescence between air bubbles at the bifurcation is observed due to the high packing density of the bubbles. Moreover, a longer time is required for the breakup after the coalescence. Besides, low interfacial tension is recommended for the symmetrical breakup. A multiphase Lattice Boltzmann method is utilized by Liu et al. [18] to study the breakup of droplets at a T-junction. The effect of surface characteristics is examined in particular. Asymmetrical breakup is observed when one of the branches of the T-junction is smooth while the other is inhomogeneous due to the contact angle hysteresis.

As mentioned above, a variety of human transportation systems are composed of multi-leveled bifurcations [21,22,23], such as human respiratory and circulatory systems, plant trunks and leaf veins, in which the behaviors of bubbles are more complicated. In natural systems, multi-leveled bifurcations usually show self-similarity between the higher-level and lower-level bifurcation structure and thus can be described by using a fractal tree-shaped configuration [24]. Compared with the single T-junction, the bifurcations are connected and coupled with each other, which requires a comprehensive study that takes into consideration the interaction between all levels of bifurcations. The flow rate in the branches is proven to be determined by the global structure of the network and the flow resistance caused by bubbles contributes to the complexity of the flow [25]. The density of the bubbles is also critical in determining the path of the bubble as well. A three-leveled hierarchical channel composed of T-junctions is designed by Hoang et al. [26] to increase the throughput of bubbles by breaking up evenly. Three sources are found to cause the asymmetrical breakup of bubbles, which are the polydispersity of the bubbles at the inlet, asymmetrical behaviors from non-break bubbles and fabrication inaccuracies of the device.

However, most results for the breakup of bubbles are obtained from experimental observations especially in systems with multi-leveled bifurcations. The behavior of bubbles splitting in bifurcation channels is usually studied in the form of a single bubble flowing in through a T-junction, especially in numerical simulations [27]. The universal condition of bubble streams flowing through a series of bifurcations is less considered. The dynamic characteristics of gas–liquid two-phase flow in the fractal tree-shaped network transportation system have not been fully understood. In particular, the interaction of fluids between different levels, the variation of pressure and the dependence between bubble volume distribution and global structure of the network is still unrevealed. Therefore, a two-dimensional numerical model of gas–liquid two-phase flow in a fractal tree-shaped network is developed based on the VOF method. The effects of fractal configuration and capillary number are discussed with detailed behaviors of the interfaces and variation of the pressure. The deformation, splitting and coalescence characteristics of the bubbles at each level of the tree branches are compared and analyzed in an effort to gain further insight into the development and evolution mechanism of distinct flow patterns. The interaction between densely packed bubbles at bifurcation is investigated comprehensively with in-depth insight of the pattern transition. Moreover, as mentioned above, the fractal tree-shaped network is highly efficient in mass distributing and the numerical simulation results are beneficial for the design of innovative drug delivery system. In addition, the influence of the feedback from the fluid flow downstream on upstream bubble dynamics is significant in the configuration of tree-shaped delivery networks.

## 2. Results and Discussions

**Figure 1:**
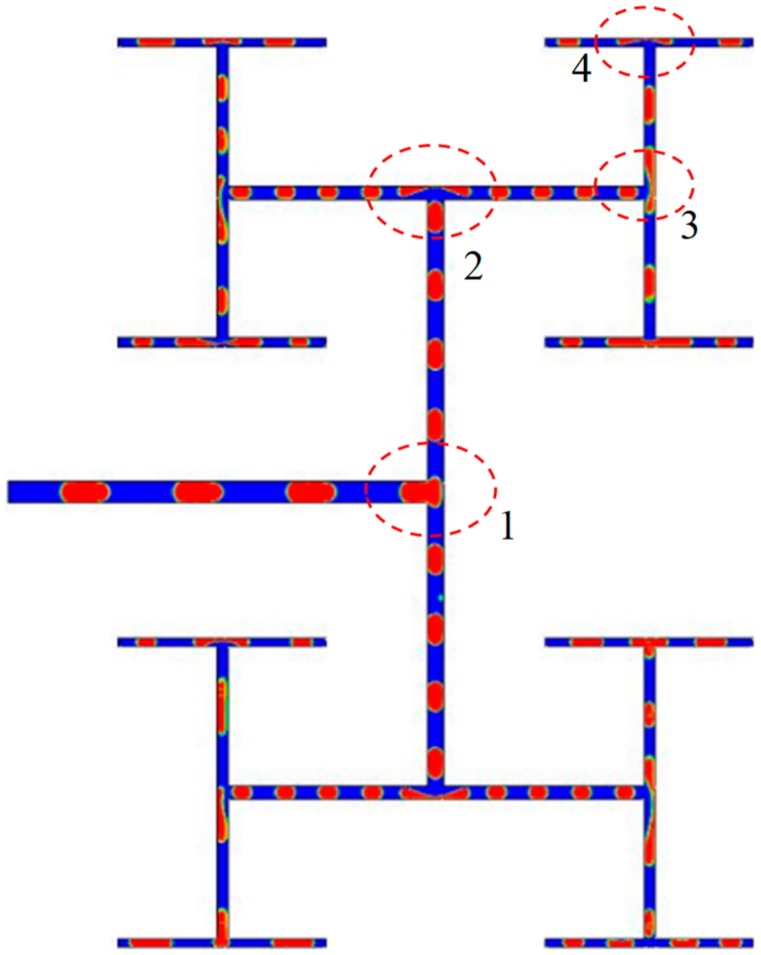
Typical case of bubble breaking up in fractal tree-shaped microchannel (Ca_0_ = 0.0111).

The fractal tree-shaped channel can be considered as a derivative from multi-leveled T-junctions and thus the bubble behaviors in fractal tree-shaped channels are similar to those flowing through a single T-junction [24]. However, the upstream and downstream fluid flow affects interactively all levels of bifurcations resulting in complicated behaviors of bubbles in the fractal tree-shaped channel as shown in Figure 1.

### 2.1. Obstructed Breakup at the First Bifurcation

**Figure 2:**
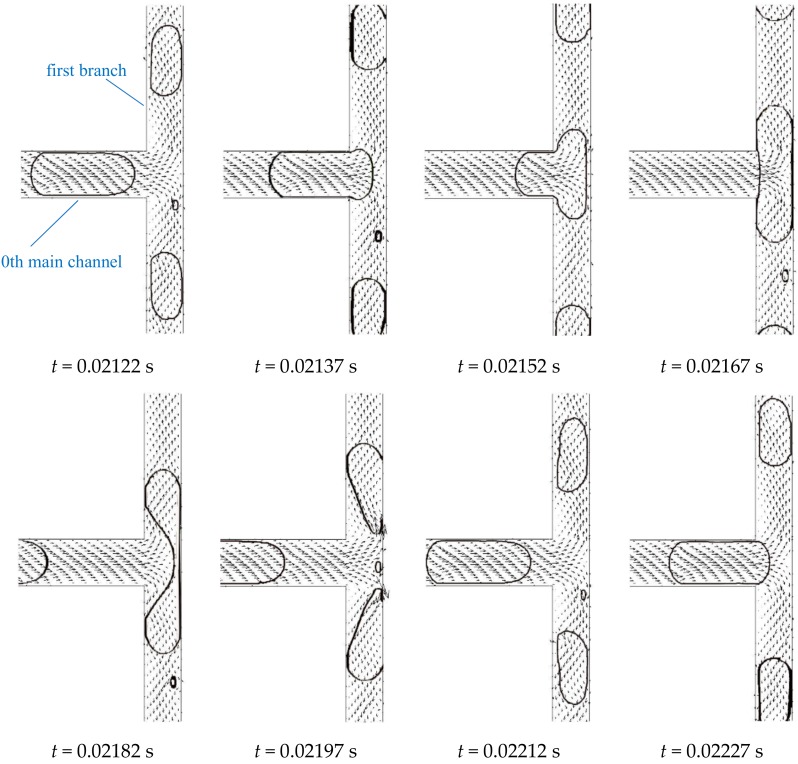
Bubble breakup at the first bifurcation of the fractal tree-shaped microchannel (Ca_0_ = 0.011).

The evolution of interface shape and velocity vectors during one bubble splitting at the first bifurcation (Figure 1) of the fractal tree-shaped microchannel is reconstructed in Figure 2. *Ca_0_* is the inlet capillary number at the 0th main channel. Since the length of the bubble is longer than the width of the channel, the bifurcation is blocked permanently by the bubble (*t* = 0.02152–0.02182 s). The bubble is split under the regime of obstructed breakup [28]. The interface is pushed by the continuous phase and bent toward the flow direction forming a round neck as shown in Figure 2 (*t* = 0.02182 s). The velocity of the continuous phase upstream of the neck decreases while pressure accumulation is observed by Chen and Deng [29] indicating that the kinetic energy is transforming into surface energy. The thickness of the neck thins over time accompanied with the gas being drained inside the neck. Eventually, the bubble breaks into two daughter bubbles and flows into the first branches of the microchannel. The average time duration of bubble splitting in the first bifurcation is 0.0006972 s (defined as the time between the front of the bubble entering the bifurcation to the instant of breaking). The velocity of both the gas and liquid is high at the back of the just ruptured interface, which helps to regain smooth interfaces indicating that the surface energy is transforming into kinetic energy.

### 2.2. Obstructed-Tunnel Combined Breakup at the Second Bifurcation

**Figure 3:**
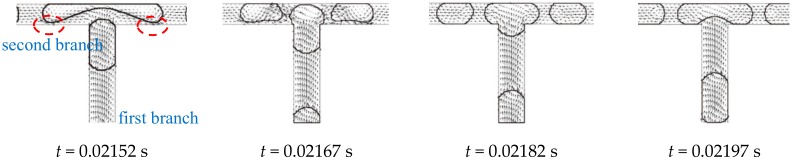

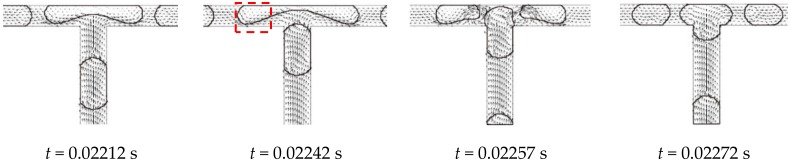
Bubble breakup at the second bifurcation of the fractal tree-shaped microchannel (Ca_0_ = 0.011).

When the daughter bubbles flow to the second level bifurcation, combined behavior of obstructed breakup and tunnel breakup [28] is observed. As shown in Figure 3, *t* = 0.02182~0.02197 s, so that the second level branches are obstructed temporarily when the bubble is entering the bifurcation. However, as the bubble is stretched along the second branches, the volume of the bubble is insufficient to fully fill the second branches. Hence, gaps are observed between the bubble and the wall as illustrated inside the dashed line in Figure 3 at *t* = 0.02152 s. The gaps act as tunnels and hence the continuous fluid is able to circumvent the bubble into the second branches through these two tunnels. Once the tunnels are opened, the velocity of the continuous fluid increases rapidly crossing the tunnel leading to a high viscous force on the bubble in the second branches. Different from the obstructed breakup, pressure upstream of the bubble is released due to the tunnel. Moreover, vortexes are observed inside the stretching lobes of the bubbles (dashed line in Figure 3 *t* = 0.02242 s) which act as an obstruction to the further deformation and elongation of the bubble according to Chen and Deng [29]. Hence, bubble splitting requires longer time compared to that in the first bifurcation. The average time required in splitting one bubble in the second bifurcation is 0.0010591 s.

As proved by the literature [28], the behaviors of bubbles at a bifurcation depend on the capillary number of the continuous phase *Ca_k_*, the length of the bubble *l_k_* and the width of the channel before bifurcation *w_k_*. Obstructed breakup occurs under the condition of high *Ca_k_* and extension of the bubble $\frac{l_{k}}{\pi w_{k}}$. While in the fractal tree-shaped channels, these dominating factors vary in each level of bifurcation. The ratio between the capillary number of the *k* + 1th branch and the *k*th branch is $\frac{Ca_{k+1}}{Ca_{k}}=\frac{v_{k+1}}{v_{k}}=\frac{Q_{k+1}}{Q_{k}}\cdot \frac{d_{k}}{d_{k+1}}=\frac{1}{2}\cdot N^{\frac{1}{\Delta }}\approx 0.63$. It is indicated that the capillary number decreases as the bubbles flow into the next level of branches. Consequently, the viscous force imposed on the bubble at the next level of bifurcation decreases as well. Since the bubble dynamics are examined in a two-dimensional configuration, the volume of the bubble can be estimated as $V_{k}=H\left[\left(l_{k}-w_{k}\right)w_{k}+\frac{\pi }{4}w_{k}^{2}\right]=H\cdot w_{k}^{2}\cdot \left[\frac{l_{k}}{w_{k}}+\left(\frac{\pi }{4}-1\right)\right]$ where *H* is the height of the channel. Assuming that the width of the bubble *w*_k_ flowing in the *k*th level of branches is the same as the width of the branch *d*_k_, the relation between the extension of a bubble in the *k* + 1th and *k*th level branch is then calculated as $\frac{l_{k+1}}{\pi w_{k+1}}=2^{-\frac{1}{3}}\cdot \frac{l_{k}}{\pi w_{k}}+\left(\frac{1}{4}-\frac{1}{\pi }\right)\cdot \left(2^{-\frac{1}{3}}-1\right)\approx 0.79\frac{l_{k}}{\pi w_{k}}+0.014$. $\frac{l_{k+1}}{\pi w_{k+1}}\geq \frac{l_{k}}{\pi w_{k}}$ only happens under the condition of $\frac{l_{k}}{w_{k}}\leq 0.27$ which is impossible for natural bubble shape inside a microchannel. The value of $\frac{l_{k}}{\pi w_{k}}$ is always larger than $\frac{1}{\pi }$ which leads to a result of $\frac{l_{k+1}}{\pi w_{k+1}}<\frac{l_{k}}{\pi w_{k}}$ unconditionally. Hence, both *Ca_k_* and $\frac{l_{k}}{\pi w_{k}}$ decrease as the bubbles flow into the higher levels of branches. This transition of the breakup regime in fractal tree-shaped channels agrees with the numerical results of Liu et al. [18] and Leshansky et al. [17]. Under the condition of *Ca*_0_ = 0.011, viscous force is still sufficient to cause symmetrical breakup at the second level bifurcation.

### 2.3. Coalesced Breakup at the Third Bifurcation

**Figure 4:**
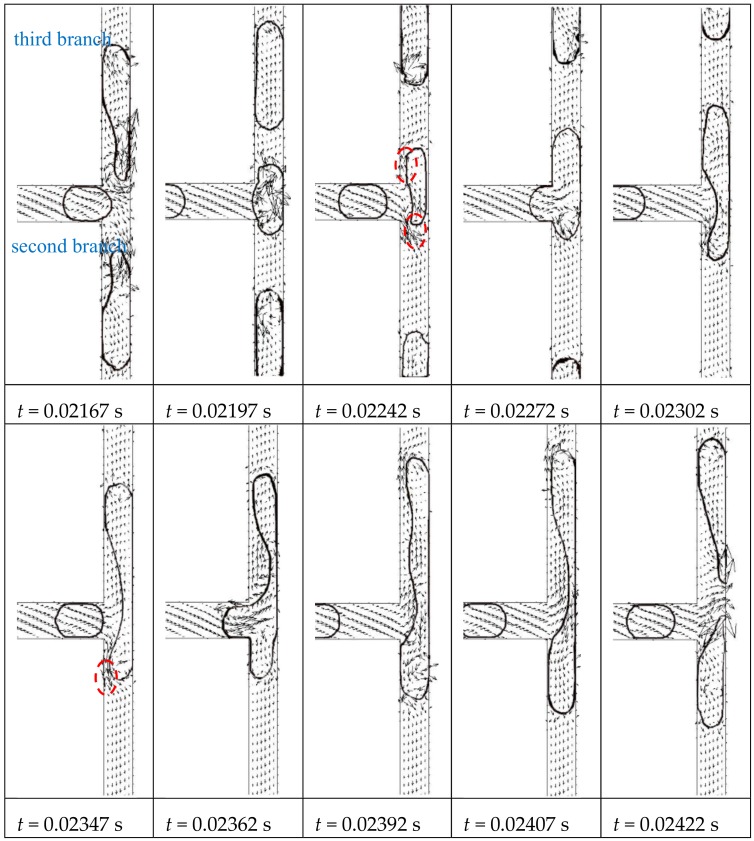
Bubble breakup at the third bifurcation of the fractal tree-shaped microchannel (Ca_0_ = 0.011)

After the symmetrical breakup at the first and second bifurcations, daughter bubbles with smaller sizes enter the third bifurcations at a lower velocity. According to Link and Fu non-break behaviors are observed when *Ca_k_* and $\frac{l_{k}}{w_{k}}$ decrease further and the threshold between breakup and non-break is $Ca_{k}=\alpha \frac{l_{k}}{w_{k}}\left({\left(\frac{l_{k}}{\pi w_{k}}\right)}^{-\frac{2}{3}}-1\right)$ [13,30,31]. However, surfactants are involved in both experimental observations and the distances between the droplets [29] or the bubbles [28] are long enough to prevent the dispersed phase from merging, whereas a stream of bubbles with short spacing between them is considered in our work to show that merging between bubbles are observed. The behavior of the first bubble agrees with the literatures [13]. The bubble stagnates at the bifurcation without breaking and flows into an arbitrary third branch due to random perturbation. Since the distances between bubbles are closer than in the previous literature [13] the continuous fluid is drained between the bubbles through the tunnels as shown inside the dashed lines in Figure 4 *t* = 0.02242 s. Moreover, the following bubble catches up with the previous bubble at the bifurcation and coalesces into a bigger bubble. Before the coalescence, the bubble is already asymmetrically distributed inside the branches and the volume difference of the bubble inside the third branches enlarges after the first coalescence. Since the capillary number is low at the third bifurcation, viscous force is insufficient to cause breakup after the first coalescence. One tunnel (dashed line in t = 0.02347 s in Figure 4) is observed in the bottom branch so that the continuous phase is drained through this tunnel and the third bubble catches up with the coalesced bubble at the bifurcation. Eventually, the bubble breaks up asymmetrically into two unequally sized daughter bubbles after two coalescences. Compared with the symmetrical behaviors of bubbles at the first and second bifurcations, the breakup of the bubbles in the third bifurcation is more irregular. Moreover, the breakup is different in the four third bifurcations in the fractal tree-shaped channel. This indicates that the flow conditions in the second branches are different, which may cause asymmetrical feedback to the outlets of second bifurcation. 

### 2.4. Pressure Variation at Different Levels of Bifurcation

**Figure 5:**
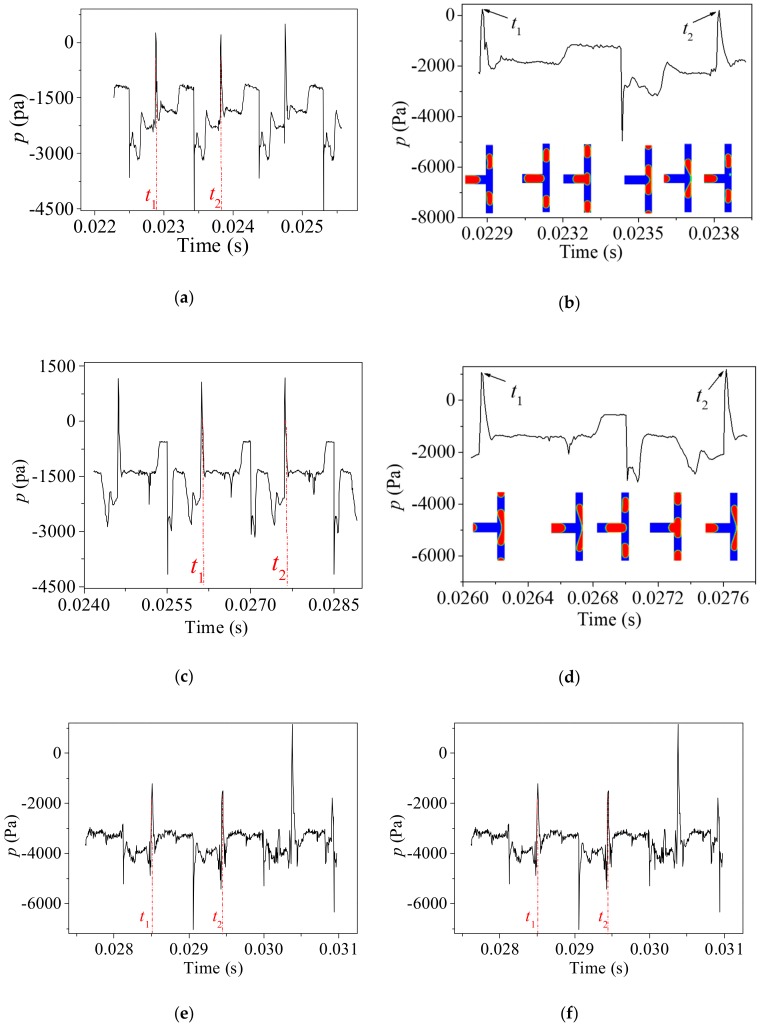
Pressure variation at the center of the first bifurcation. (**a**,**b**): *Ca_0_* = 0.0111; (**c**,**d**): *Ca_0_* = 0.00694; (**e**,**f**): *Ca_0_* = 0.00416.

The pressure of the continuous phase upstream from the bubble at the bifurcation is essential during the breakup of bubbles [29]. Moreover, insufficient pressure leads to non-break behavior of bubbles [29]. Figure 5a gives the gauge pressure variation with time in several periods of bubble breakup at the center of the first bifurcation under the condition of *Ca_0_* = 0.0111 so that the bubble undergoes obstructed breakup. As shown in the figure, the pressure varies periodically with a period *T* = *t*_2_ – *t*_1_ = 0.00094 s. To explore the pressure characteristics in one time period, the pressure and morphology evolutions of the bubble interface are shown in Figure 5b. *t*_1_ and *t*_2_ are the moments when the bubble is breaking up, and the pressure of reaches the maximum value during the whole breakup process. Initially, the pressure is relatively stable, resulting from the fact that the center of the bifurcation is filled with the continuous phases. As the bubble head enters the bifurcation, the pressure rises to a relatively high value and then fluctuates in a small amplitude. Afterward, the bubble head reaches the front wall of the channel and a sudden pressure drop is observed due to the stretching of the bubble inside the first branches. When the back of the bubble enters the bifurcation, the branches are obstructed by the bubble and hence a pressure increment is observed. During this period of time, the viscous force is competing with the interfacial tension and the curvature of the bubble back is varying with time. Hence the pressure is fluctuating at a bigger amplitude until the bubble breaks up.

Figure 5c,d illustrates the evolution of the pressure at the center of the first bifurcation under the condition of *Ca_0_* = 0.00694 that the bubble undergoes a tunnel breakup regime. While the pressure variation follows a similar tendency as the obstructed breakup shown in Figure 5a, the behaviors of the bubbles are different. The pressure at the bifurcation reaches the maximum at the instant of tunnel opening as illustrated in the insets of Figure 5d. After that, the pressure upstream from the bubble is released due to the draining of the continuous fluid through the tunnel. The duration time of a circle is 0.001504 s which is longer than that of the obstructed breakup.

As the capillary number decreases further to 0.00416, the viscous force is insufficient to cause the breakup of the first bubble and the coalescence breakup occurs even at the first level of bifurcation as shown in Figure 5e,f. Similar to the tunnel breakup, the pressure at the center of bifurcation reaches a maximum value (*t*_1_ in Figure 5f) when the tunnels are opened between the first bubble and channel walls. After the coalescence, the pressure at the center of the first bifurcation decreases drastically due to the bubble stretching inside the branches. Since the branches are obstructed by the coalesced bubble, the pressure reaches another maximum (*t*_2_ in Figure 5f) at the instant of the bubble breaking up. The duration of time of one-time coalescence break up under the condition of *Ca_0_* = 0.00416 is 0.002502 s. Generally, the bubbles are subjected to weaker viscous force under a smaller capillary number. As a result, the breakup of bubbles requires a longer time.

**Figure 6:**
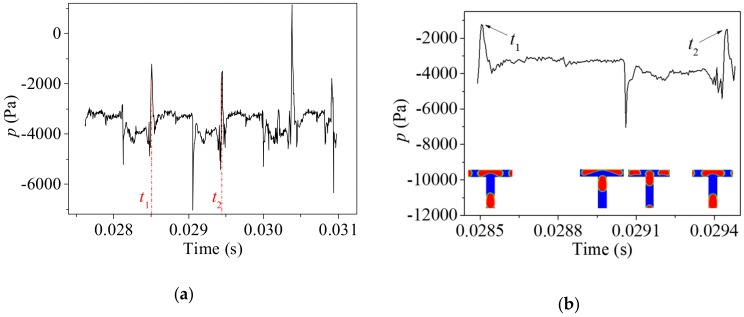

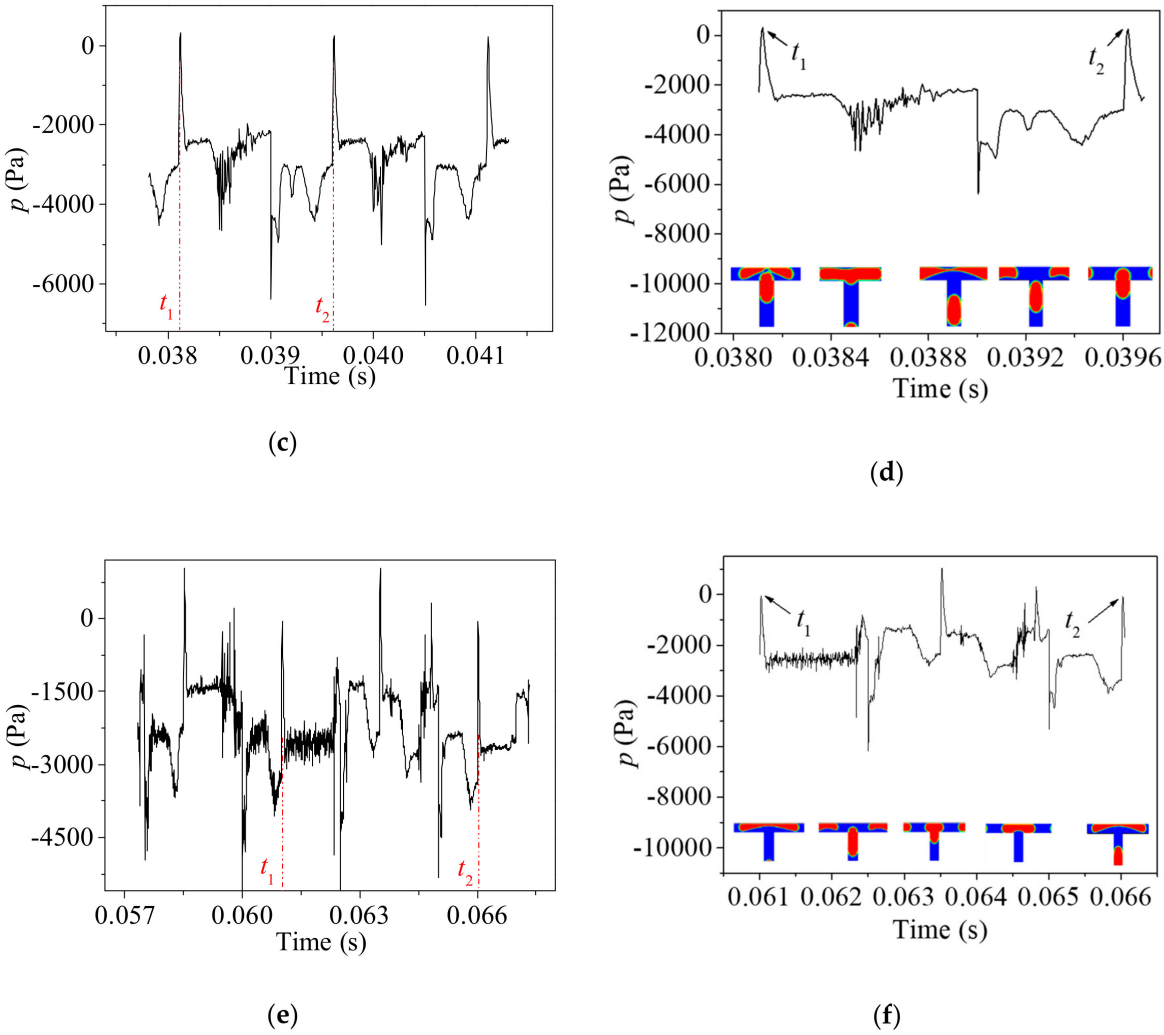
Pressure variation at the center of the second bifurcation. (**a**,**b**): *Ca_0_* = 0.0111; (**c**,**d**): *Ca_0_* = 0.00694; (**e**,**f**): *Ca_0_* = 0.00416. (Pressure variation during the selected time period from the left column are shown in the right column. *t*_1_ and *t*_2_ are the beginning and end of the selected time period, respectively as the arrows pointed in the right column).

Figure 6 compares the variation of the pressure at the center of the second bifurcation under the different value of the inlet *Ca_0_*. Tunnel breakup without the coalescence (Figure 6a,b) is observed at the second bifurcation when *Ca_0_* = 0.0111. The behavior of the bubble and the variation of pressure is similar to the tunnel breakup shown in Figure 5c,d. Figure 6c,d shows the evolution of pressure at the center of the second bifurcation at a smaller capillary number *Ca_0_* = 0.00694 when coalescence is observed. The pressure reaches its maximum value and decreases sharply when the two bubbles coalesce at the bifurcation and fluctuations are observed several times during a cycle. Attributed to the symmetrical breakup in the first bifurcation, the breakups in the second bifurcation under the condition of *Ca_0_* = 0.0111 and *Ca_0_* = 0.00694 are still periodic. On the other hand, unequally sized bubbles are produced in the first bifurcation asymmetrically under *Ca_0_* = 0.00416. Hence, no strict periodicity is observed at the second bifurcation. The pressure variation fluctuates irregularly without obvious periodicity due to the complex behavior of bubble upstream in the first bifurcation.

**Figure 7:**
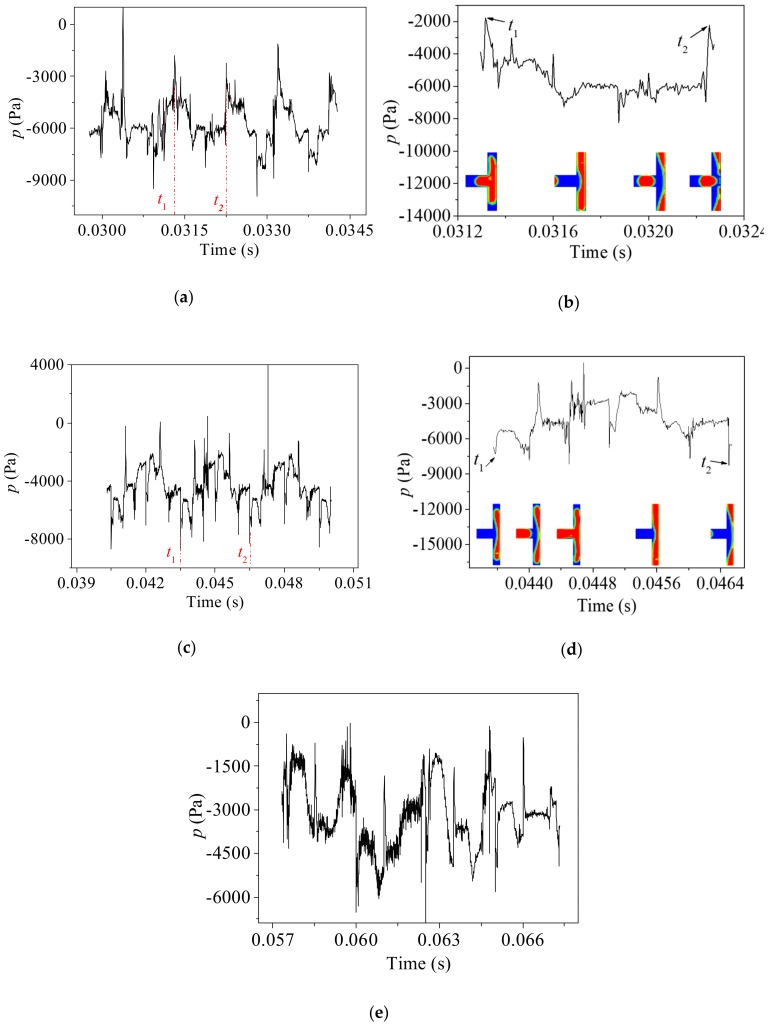
Pressure variation at the center of the third bifurcation. (**a**,**b**): *Ca_0_* = 0.0111; (**c**,**d**): *Ca_0_* = 0.00694; (**e**): *Ca_0_* = 0.00416.

As stated above, the behavior of bubbles at the upstream bifurcation has a strong influence on the behaviors of bubbles at the downstream bifurcations. Figure 7 summarizes the pressure variation at the center of the third bifurcation under different capillary numbers. Compared with the first and second bifurcations, the pressure fluctuation at the third bifurcation is more complex and less periodic, which indicates that the flow pattern and velocity distribution at the third bifurcation is merely random.

Under a relatively high inlet capillary number *Ca_0_* = 0.0111, the bubble length and viscous force at the third bifurcation is still insufficient to cause the breakup of the first bubble. As illustrated in Figure 7a,b the pressure rises and drops sharply due to the coalescence of the bubbles at time *t*_1_. Then the bubble enters into and the stretching at the bifurcation resulting in a decrement in the pressure gradually. After the gas–liquid interface of the bubble neck crosses the center of the bifurcation, the center is filled with gas phase and the pressure fluctuates at a finite value. Moreover, finally, the bubble breaks up, leading to a sudden increase in the pressure. Weak periodicity of pressure variation is observed in Figure 7a. The fluctuation of the pressure becomes more drastic as shown in Figure 7c,d at a smaller inlet capillary number of 0.00694. As the viscous force acting on the bubble weakens further with the decreasing *Ca_0_*, the bubbles are less likely to break and the fluctuation of the pressure is harder to suppress by bubble breakup.

**Figure 8:**
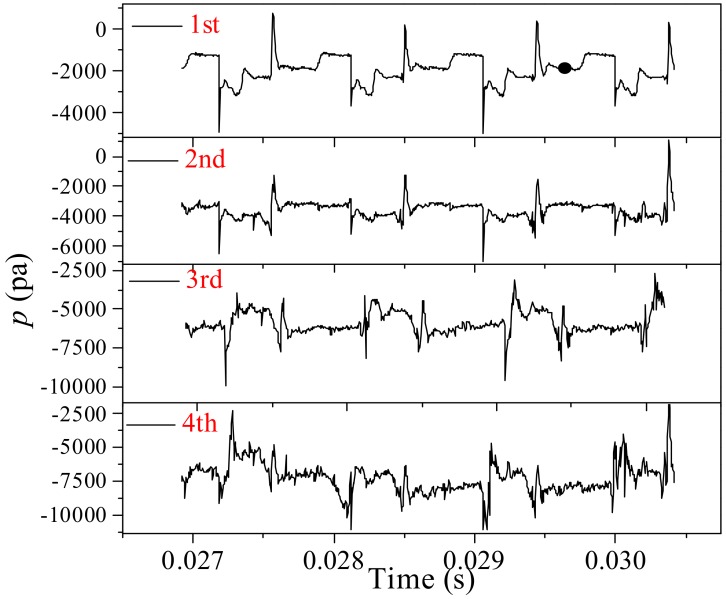
The effect of a fractal structure on the time evolution of the pressure at the center of each bifurcation (*Ca_0_* = 0.0111).

Comparing Figure 5, Figure 6 and Figure 7, it is indicated that the behaviors of the bubble become more irregular when flowing into a higher level of branches. To further explore the characteristics of the bubble breakup in the fractal tree-shaped channel, comparisons of the pressure at the center of each bifurcation are summarized in Figure 8. At the first and second bifurcation, the pressure variation shows obvious periodicity. Notably, the difference between the duration time of each period in the same bifurcation is below 1%. However, compared with the first bifurcation, the fluctuation of pressure is more obvious at the second bifurcation, particularly during the process of bubble stretching in the bifurcation. As stated above, tunnel breakup is observed at the second bifurcation and the continuous fluid circumvents the bubble during the stretching process. The flow of the continuous fluid is not so segmented that the flow field is affected by the downstream feedback from the third bifurcation [32], while at the third bifurcation, the fluctuation is further magnified so that the periodicity weakens. In addition, both the velocity and size of the bubble are reduced in the higher-level bifurcations. The bubbles are less likely to break up in the higher-level bifurcation, which gives rise to erratic coalescence between bubbles. Moreover, the non-break bubble before coalescence moving into the arbitrary branches also leads to unbalanced pressure between the branches. Consequently, more irregular behavior of bubbles in the higher-level channels occurs. At the fourth bifurcation, no priority of the pressure variation is observed, which indicates completely random behaviors of the bubbles. To sum up, fluctuations in the flow filed at the lower level bifurcation propagate with the flow and magnify successively at each level of the fractal tree-shaped channel. As a result, chaotic pressure variation is observed at the high-level bifurcations. On the other hand, the continuous phase is not segmented permanently, the downstream pressure distribution acts as a feedback to the upstream bifurcation. As a result, the breakup of the bubbles at the upstream bifurcations are subject to unequally distributed pressure outlets that can increase the possibility of asymmetrical behavior of the bubbles upstream. Therefore, the interaction between the different levels of bifurcations in the fractal structure results in the complex behavior of bubbles and magnifies perturbation in the flow filed as the bubbles flowing into higher levels of bifurcations.

## 3. Mathematical Model

**Figure 9:**
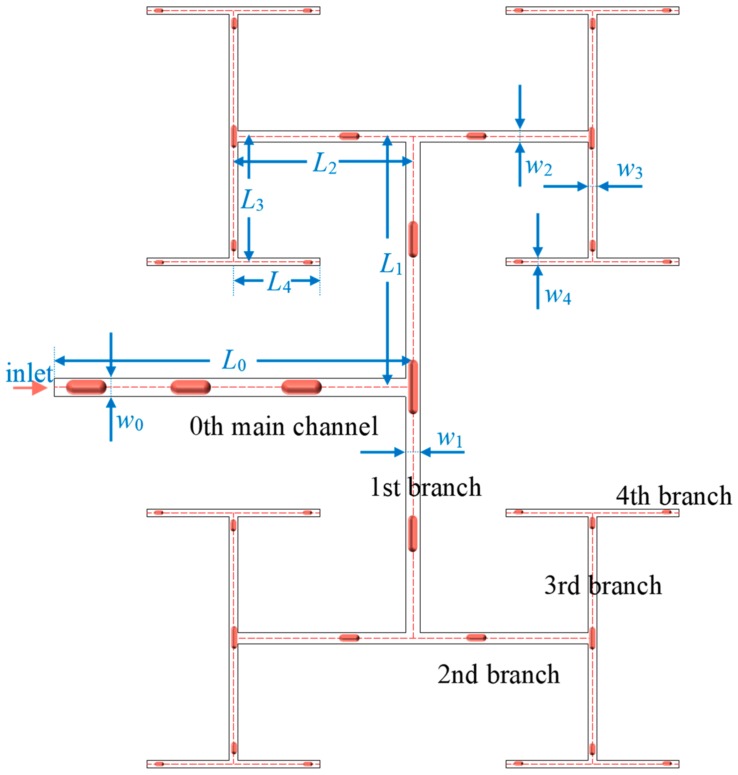
Tree-shaped microchannel.

A two-dimensional numerical model is developed to study the behaviors of bubble stream flow in a tree-shaped microchannel as demonstrated in Figure 9. *L*_0_–*L*_4_ are the channel lengths from level zero to level four, respectively and the corresponding diameters of the channels are denoted as *D*_0_–*D*_4_. The dispersed phase (gas) and the continuous phase (liquid) flow into the channel from the same inlet alternatively at level zero. The flow duration of each phase is determined by a user-defined function (UDF). Using this strategy, gas bubbles are formed in situ at the inlet under the effect of interfacial tension as shown in Figure 9. The tree-shaped fractal network is generated following:

(1) $$\frac{L_{k+1}}{L_{k}}=N^{-1/D}$$

where *L_k_* is the length of the channel at level *k* and the length of the main channel is *L*_0_ = 3000 μm, *N* = 2 is the number of the branch at each bifurcation and *D* = 2 is the fractal dimension of length. The relationship between the width of the *k*th and *k* + 1th level branch can be expressed as:

(2) $$\frac{w_{k+1}}{w_{k}}=N^{-1/\Delta }$$

where Δ = 3 is the diameter of the fractal dimension and the diameter of the main channel is *w*_0_ = 1500 μm.

The volume of fluid (VOF) interface tracking method is utilized to study the evolutionary process of the Newtonian, incompressible and immiscible gas–liquid two-phase flow in the tree-shaped fractal microchannels. In the VOF method, the proportion of gas or liquid is represented by its volume fraction function *a* and *a* of all fluids sums to 1 in a computational cell:

(3) $$a_{g}+a_{l}=1$$

in which the subscripts g and l denote gas and liquid, respectively.

The transport equation of the volume fraction of gas is:

(4) $$\frac{\partial a_{g}}{\partial t}+\vec{U}\nabla a_{g}=0$$

where *t* is the flow time and $\vec{U}$ is the velocity which can be obtained from the continuity equation:

(5) $$\nabla \cdot \vec{U}=0$$

and the Navier-Stokes equation:

(6) $$\frac{\partial \vec{U}}{\partial t}+\nabla \cdot \left(\vec{U}\vec{U}\right)=-\frac{\nabla p}{\rho }+\frac{\mu }{\rho }\nabla \cdot \left[\nabla \vec{U}+\nabla {\vec{U}}^{T}\right]+F$$

where *p* is the pressure, *F* is the source term, *ρ* is the density and *μ* is the viscosity.

In the VOF method, only one set of the governing equation is solved despite the multiphase flow. The physical properties in the governing equations in one computational cell are interpolated using the volume fraction of each fluid as

(7) $$\rho =a_{g}\cdot \rho_{g}+a_{l}\cdot \rho_{l}$$

(8) $$\mu =a_{g}\cdot \mu_{g}+a_{l}\cdot \mu_{l}$$

The source term in Equation (6) is composed of two terms, the gravity *g* and interfacial tension *F*_s_. In our work, the gravity is assumed to be vertical to the plane of the channel and is therefore neglected. The interfacial tension is induced into the Navier-Stokes equation as a body force following the continuum surface force (CSF) method [33] as:

(9) $$F_{s}=\sigma \kappa \hat{n}\delta_{s}$$

where *σ* is the interfacial coefficient, $\hat{n}=\frac{n}{\left|n\right|}$ is the unit normal to the interface, $\kappa =\nabla \cdot \hat{n}\;i\;$s the curvature of the interface and *δ*_s_ is the delta function.

The bubble dynamics are governed by the viscous force from the continuous phase and the interfacial tension. Moreover, the capillary number is used to measure the relation between the viscous force and interfacial tension

(10) $$Ca_{k}=\frac{\mu_{l}v_{k}}{\sigma }$$

where *v*_k_ is the velocity of the liquid phase at the *k*th level of branch.

The velocity inlet boundary is set as the inlet boundary condition of the computational domain with a user-defined function (UDF) defining the flowing duration of gas and liquid. Pressure outlet boundaries are set at all 16 outlets of the branches. Moreover, a non-slip boundary condition is adopted at the wall of the tree-shaped channel.

**Figure 10:**
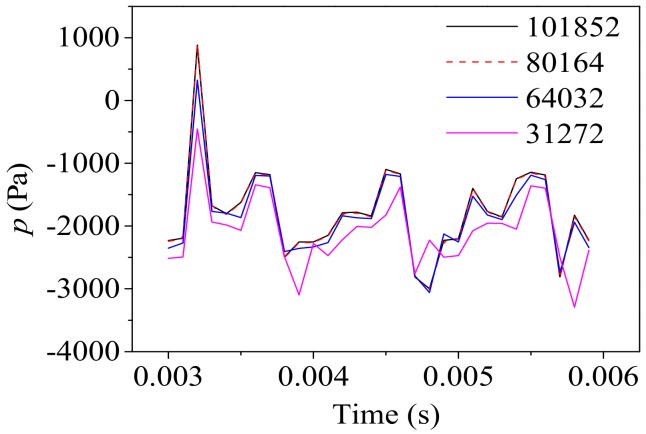
Mesh independence analysis.

The computational domain is discretized using a square structured grid. A mesh independence analysis is conducted and the gauge pressure fluctuation of the same monitoring point (the center at the first bifurcation) under the same boundary conditions calculated using different grid numbers are compared (Figure 10). It can be concluded that the results obtained from the mesh with grid numbers of 80164 and 101852 are in good agreement. Considering both the convergence of the calculation and the computational cost, mesh with the grid number of 80164 is used in this paper. 

**Figure 11:**
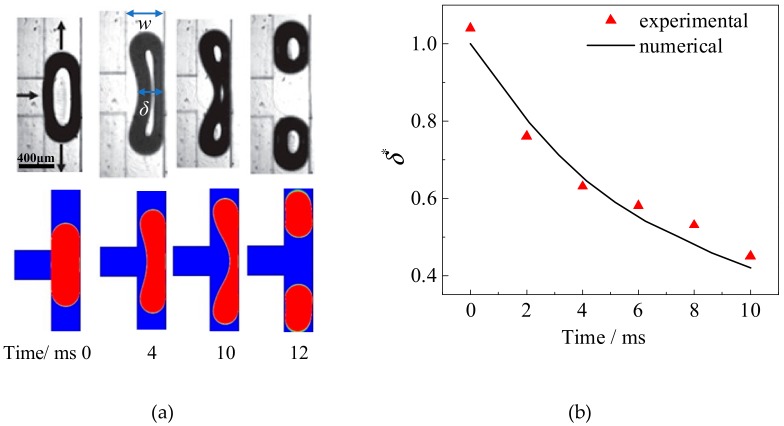
Comparison of experimental and numerical results of bubble breakup in a T-junction. (**a**) Evolution of the bubble shape, (**b**) variation of neck thickness with time [13].

Besides, the behaviors of the bubbles obtained from numerical simulation are compared with experimental observation [13] to verify the mathematical model as presented in Figure 11. The evaluation of the interface shape (Figure 11a) during the stretching of the bubble at the bifurcation reconstructed from numerical results agrees well with the experiment. In addition, the variation of the dimensionless neck thickness (δ* = *δ*/*w*) is compared quantitatively as summarized in Figure 11b. The agreement between numerical simulation and experimental results indicates that the numerical model developed in our work is capable of predicting the dynamic behaviors of the bubbles flowing through the tree-shaped microchannels comprised by a series of T-junctions.

## 4. Numerical Solution

Since the fluid in flow is considered in a microchannel and the typical Reynold number is smaller than 10, the laminar model is implemented in the simulation. The finite volume format is adopted when solving the partial differential equations. The explicit scheme is utilized for the VOF equation (Equation (4)). The body force weighted format is used for the pressure interpolation. The second-order upwind format is used for the discretization of the momentum equation. Moreover, the coupling between the pressure and velocity is achieved through the semi-implicit method for pressure linked equations (SIMPLE) algorithm. In order to acquire convergence, underrelaxation factors are used for some parameters in the governing equation, which are 0.3 for the pressure term, 0.3 for the density term, 0.3 for the volume force source term and 0.2 for the momentum source term. The time step is varied following the criterion that the global Courant number does not exceed 0.5. The magnitude of the time step is basically 10^−6^ s. The convergence standard is achieved when the relative difference between iterations is below 1%.

## 5. Conclusions

In this paper, a two-dimensional numerical model of gas–liquid two-phase flow in a fractal tree channel is developed to investigate the hydrodynamic behavior of bubble streams. The evolutions of the flow field and gas–liquid interface are examined to study the transition of breakup regimes as bubbles flow into higher levels of bifurcations. In an effort to clearly understand the underlying mechanism of bubble breakup, the pressure evolution at the center of different levels of bifurcation under different inlet capillary numbers is discussed. The results indicate that:

(1) Under a constant inlet capillary number, the lengths of bubbles and local capillary numbers decrease when the bubbles are flowing into higher levels of bifurcations. Moreover, the breakup regime of the bubbles transit from the obstructed breakup and obstructed-tunnel combined breakup to coalescence breakup. 

(2) Periodicity in the pressure variation can only be observed for the first and second bifurcation under the condition of high inlet capillary numbers. Decreases in the inlet capillary number lead to coalescence of bubbles at the first bifurcation and fluctuation of pressure in all levels of bifurcation.

(3) With the same capillary number, the pressure variation at the center of the bifurcation becomes more disordered with the increase of the fractal levels. The perturbation in the lower level channel is magnified by the tree-shaped channel and the fluid flow becomes more chaotic when flowing into higher levels of branches.

A fractal tree-shaped channel is efficient in distributing mass in a low-cost approach, which can be beneficial for the design of innovative drug delivery networks using bubbles as delivery vessels.
